# Systemic reactions to subcutaneous allergen immunotherapy: real-world cause and effect modelling

**DOI:** 10.1186/s13223-021-00566-x

**Published:** 2021-07-06

**Authors:** Adam Aue, Joella Ho, Rongbo Zhu, Harold Kim, Samira Jeimy

**Affiliations:** 1 Faculty of Medicine, University of Ottawa, Ottawa, ON Canada; 2 Schulich School of Medicine and Dentistry, Western University, London, ON Canada; 3 Division of Clinical Immunology and Allergy, Department of Medicine, Western University, London, ON Canada; 4 Department of Medicine, McMaster University, Hamilton, ON Canada; 5 St. Joseph’s Healthcare London, B3-112, 268 Grosvenor Street, London, ON N6A 4V2 Canada

**Keywords:** Systemic reaction, Anaphylaxis, Quality improvement, Subcutaneous immunotherapy, Immunotherapy, Aeroallergen, Allergy

## Abstract

**Background:**

Subcutaneous immunotherapy (SCIT) is an effective treatment for allergic rhinoconjunctivitis. However, adverse events, including life-threatening systemic reactions, may occur. The purpose of this project is to identify risk factors for systemic reactions to SCIT and to provide practice-based solutions using a quality improvement (QI) framework.

**Methods:**

A QI initiative was performed in a hospital-based, Canadian Allergy clinic administering SCIT in a 12-month period.

**Results:**

A total of 4242 injections of SCIT were performed over a period of 12 months. Of these, 10 injections resulted in a systemic reaction requiring epinephrine administration (i.e., an incidence of 1 in 424 injections, or 0.24%). Eight patients had at least one documented risk factor for a systemic reaction, and six had multiple risk factors. Major risk factors included seasonal exacerbation of allergic rhinitis, uncontrolled asthma, and an error in route of administration. All reactions occurred with the highest allergen extract concentration.

**Conclusion:**

This QI initiative highlights the need for improved patient and health care practitioner education and pre-administration screening. We suggest several considerations for SCIT administration: provide patients with written information on safety; screen patients before injections, including a review of treatment plan adherence and asthma control; adjust dosing to slow down buildup of the most concentrated immunotherapy extract, particularly in high risk patients; and apply additional safety measures in patients with multiple risk factors.

## Background

Subcutaneous immunotherapy (SCIT) offers effective disease modifying therapy for several common allergic conditions. The safety and efficacy of SCIT has been established in the management of allergic rhinitis, allergic conjunctivitis, allergic asthma, and stinging insect hypersensitivity [1]. SCIT may also be considered in patients with atopic dermatitis with aeroallergen sensitization [1, 2]. The clinical utility of SCIT has been demonstrated over the years; however, the potential for adverse reactions remains a concern. Adverse reactions may present as a localized reaction at the injection site or may present systemically [3]. Systemic reaction are rare when following a conventional protocol, with a prevalence of less than 1.0% of patients and 0.1% of injection visits [4]. Systemic reactions are significantly more prevalent when using a “rush protocol” for aeroallergens, which aim to achieve maintenance dosing within 4 h to 6 days [2]. Some studies have described the prevalence of systemic reactions in rush immunotherapy to be greater than 34% of patients (in the absence of premedication) [2]. For all protocols, systemic reactions occur more frequently during the build-up phase compared to the maintenance phase [5]. Fatal reactions to SCIT are rare, with surveys reporting a rate of 1 fatal reaction per 9.1 million injection visits [4].

International societies have sought to develop a consensus on the use of allergen immunotherapy in clinical practice, with landmark contributions from the American Academy of Allergy, Asthma, and Immunology (AAAAI), and the European Academy of Allergy and Clinical Immunology (EAACI) [2, 6]. These reports include a summary of the current state of allergen immunotherapy, with attention to the risk of systemic reactions. Additionally, the 2016 Immunotherapy Manual published by the Canadian Society of Allergy and Clinical Immunology (CSACI) summarizes several risk factors, including uncontrolled asthma and/or an FEV1 < 70% predicted on spirometry, previous history of systemic reactions to SCIT, SCIT injections from a new extract vial, concomitant treatment with beta-blockers, and administration errors such as an intramuscular injection or a dosing error [1]. Risk factors should be monitored in all patients receiving SCIT. In particular, beta-blockers should be discontinued in most cases prior to starting aeroallergen SCIT, and asthma should be optimally controlled at the time of each injection. The AAAAI, EAACI and CSACI outline additional strategies to reduce the risk of anaphylaxis, including routine screening for symptomatic asthma, avoiding immunotherapy injections in patients with a respiratory infection, fever, or severe exacerbation of allergic rhinoconjunctivitis symptoms, avoiding dose increases and/or considering decreasing doses during peak allergen season, and avoiding strenuous exercise for at least 1 h before and 2 h after injection [1, 2, 6].

The implementation of these considerations requires thoughtful coordination between the patient, physician, and clinic staff. Given the risk of adverse reactions, optimizing safety in patient care is of paramount concern for all clinicians.

Limited Canadian data is available regarding the incidence of systemic reactions in SCIT and the risk factors associated with them. Thus, the purpose of this quality improvement (QI) project is to describe and identify causes of systemic reactions to SCIT requiring epinephrine administration in a hospital-based Allergy and Clinical Immunology practice and provide a quality improvement framework to mitigate such risks for patients on allergen immunotherapy.

## Methods

A QI initiative was conducted at a large Canadian academic Allergy and Immunology clinic from January–December 2019. A total of 4242 subcutaneous injections were administered during this time. A total of 860 patients were prescribed SCIT in this period, this includes both patients who received injections in our clinic, as well as those who went on to receive injections elsewhere.

As per clinic policy, prior to commencing SCIT, all patients signed informed consent and contractual agreements to adhere to the treatment plan, including the avoidance of known contraindications and risk factors that may increase the risk of adverse reactions [1]. Patients received SCIT in the clinic and were asked to remain in the clinic or hospital for at least 30 min in order to monitor for adverse reactions. Extracts for SCIT were prepared and provided by the local hospital pharmacy at St. Joseph’s Healthcare, London, Ontario, Canada. Systemic reactions were managed according to standard of care for anaphylaxis [1].

For patients who underwent immunotherapy for aeroallergens, SCIT was administered according to the protocol outlined by the CSACI Immunotherapy Manual [1]. Patients who underwent immunotherapy for stinging insect hypersensitivity followed the protocol shown in Table 1.

**Table 1:** Dosage schedule for insect venom allergen immunotherapy (VIT)

VISIT# (q1-2wk)	Conc. (mcg/mL)	Volume (mL)
1	10	0.10
	100	0.05
	100	0.10
2	100	0.15
	100	0.15
3	100	0.30
	100	0.30
4	100	0.50
	100	0.50

As part of the quality control initiative, all patient data with a systemic reaction to allergen immunotherapy requiring epinephrine administration were recorded including patient characteristics (age, sex, past medical history, risk factors, and medications) and details of the reaction (severity, presenting symptoms, reaction month and pollen count, allergens, time course of the reaction, and treatments). Dose and vial number were also documented. Systemic reactions were graded using the World Allergy Organization (WAO) SCIT Systemic Reaction Grading System [7].

## Results

During the 12-month period of the QI initiative (January–December 2019), a total of 4242 allergen immunotherapy injections were administered. Ten patients experienced systemic reactions that required epinephrine administration. The incidence of systemic reactions requiring epinephrine was 1 in 424 injections (0.24%) in this clinic patient population.

Reaction details and patient characteristics are summarized in Table 2. Patient ages ranged from 17–52 years (median age: 30.5). Most patients (80%) were female. Atopic comorbidities included: asthma (50%), eosinophilic esophagitis (10%), and food allergies (20%). Reaction timing was compared with local pollen counts; three patients received injections coinciding with moderate to high pollen counts (Table 2). Other risk factors include the use of concomitant medications, including the use of an angiotensin converting enzyme (ACE) inhibitor on the day of the injection by one patient (10%), who had also taken a non-steroidal anti-inflammatory drug (NSAID) the day prior to the injection. All of the reactions occurred with aeroallergens; as well, all occurred at the highest concentration of allergen extract (vial 4). The injection volumes ranged from 0.05–0.50 mL. Five reactions (50%) occurred in the build-up phase, including three patients (30%) who reacted while receiving 0.05 mL of extract from vial 4, and five reactions (50%) occurred in the maintenance phase (0.5 mL of vial 4). Lastly, all reactions occurred with extracts containing multiple allergens (lowest number of allergens per extract: two; highest: seven). The majority of reactions (80%) occurred with vials containing a mixture of standardized and non-standardized extracts, and a minority contained standardized extracts only (20%).

**Table 2:** Characteristics for patients with systemic reactions to SCIT

Patient number	Age	Gender	Relevant comorbidities	Reaction grade (WAO)	Time to reaction (min)	Pollen prevalence	Extract content	Extract volume (all patients were on 1:1 concentration of extract)	Risk factor
1	23	Female	None	3	5	None	1st vial 1. Dust mite 2. Cat 2nd vial 1. Mixed tree 2. Grass 3. Ragweed	0.3 mL	1. Accidental omission of pre-mediation (Rupatadine, 10 mg, PO) 2. First injection from a new serum vial
2	22	Female	None	2	60	None	1. Cat 2. Ragweed	0.5 mL (maintenance)	High intensity exercise 1 h prior to injection
3	52	Male	Asthma	2	5	Grass (high), Walnut (high), Tree (high)	1st vial 1. Dust mite 2. Cat 2nd vial 1. Grass 2. Ragweed 3. Birch 4. Walnut	0.15 L	1. Seasonal exacerbation: a build-up dose that included walnut, grass, and tree allergens was given during peak pollen season 2. ACE inhibitor (Perindopril) was taken the day of the injection 3. NSAID (Naproxen) was taken the day prior to the injection 4. Previous large local reaction
4	17	Female	None	2	15	Grass (low; high the prior week)	1. Dust mite 2. Cat 3. Ragweed 4. Grass	0.1 mL	1. First injection from a new serum vial 2. History of previous systemic reactions to SCIT 3. Seasonal exacerbation: a build-up dose was given during moderately high grass pollen season
5	25	Female	Asthma	2	30	Grass (low), Cladosporium (high)	1. Dust mite 2. Cat 3. Cladosporium 4. Grass 5. Black walnut	0.5 mL	1. History of previous systemic reaction to SCIT 2. Seasonal exacerbation: a maintenance dose coincided with high cladosporium counts
6	37	Female	Asthma	2	15	Trees (medium), weeds (low)	1st vial 1. Dog 2. Cat 3. Alternaria 2nd vial 1. Grass 2. Ragweed 3. Birch 4. Elm	0.05 L	1. Uncontrolled asthma 2. First injection from a new serum vial 3. Error in administration suspected: the injection site did not appear subcutaneous 4. Seasonal exacerbation: a build-up dose was given during peak pollen season
7	33	Female	Asthma	2	40	Grass (low)	1st vial: 1. Dust mite 2. Cat 3. Dog 2nd vial 1. Grass 2. Ragweed 3. Birch 4. Oak 5. Walnut 6. Box elder 7. Sycamore	0.5 mL	1. History of previous systemic reactions to SCIT 2. Uncontrolled asthma
8	33	Male	None	1	*Unknown, time not documented*	None	1. Box elder 2. Birch 3. Sycamore 4. Mulberry 5. Walnut 6. Grass 7. Ragweed	0.5 mL	None
9	29	Female	None	1	5	None	1. Dust mite 2. Grass 3. Ragweed 4. Walnut	0.2 mL	None *Note: This patient was experiencing longstanding respiratory and cardiac symptoms of unknown etiology at the time of the reaction*
10	32	Female	Asthma	1	15	None	1st vial 1. Box elder 2. Birch 3. Walnut 4. Grass 5. Ragweed 2nd vial 1. Dust mite 2. Cat	0.1 mL	First injection from a new vial

*ACE* Angiotensin converting enzyme, *NSAID* Nonsteroidal anti-inflammatory drug, *PO* Per os (by mouth), *SCIT* Subcutaneous immunotherapy, *WAO* World Allergy Organization

Extract composition: Dust mite extract includes D. Pteronyssinus and D. Farinae. Timothy grass was used for Grass extract. Tree extracts included a mix of relevant pollens for Southwestern Ontario, Canada

Seven patients (70%) reacted within 30 min of the injection. Two patients (20%) experienced a delayed reaction onset, one occurring 40 min, and the second 60 min post-injection. Time to reaction onset was not documented for one patient. According to the WAO grading system, three reactions (30%) were grade one, six reactions (60%) were grade two, and one (10%) reaction was grade three. There were no grade four reactions, and no reactions were fatal.

We identified multiple risk factors in patients with a systemic reaction to allergen immunotherapy requiring epinephrine administration. Six patients (60%) had several potential risk factors that included: accidental omission of pre-medication, injection from a new vial despite appropriate dose reduction, injection during high pollen season, concomitant ACE inhibitor use, uncontrolled asthma, possible error in administration route, and previous history of systemic reactions to SCIT. There were two patients (20%) with only a single risk factor: one patient received an injection from a new vial (without dose reduction) and one patient reported strenuous exercise prior to injection. Two patients (20%) did not have any obvious risk factors for their reaction; however, one patient was experiencing longstanding respiratory and cardiac symptoms of unknown etiology at the time of their reaction.

All patients received epinephrine 0.5 mg (1:1000 concentration) to the anterolateral thigh. Three patients (30%) received more than one dose of epinephrine. Nine patients (90%) received epinephrine intramuscularly. One patient (10%) who experienced a mild systemic reaction received epinephrine subcutaneously, at the discretion of the supervising physician. Following epinephrine administration, eight patients (80%) remained in-clinic for observation, one (10%) was transferred to the Emergency department, and one (10%) was transferred to the Urgent Care department (following a Grade 3 reaction). Additional treatments received by patients included Salbutamol, Diphenhydramine, Cetirizine, Ranitidine, and Prednisone.

Following the adverse reactions, each patient was seen on a follow-up visit to discuss their interest in and candidacy for continuing allergen immunotherapy. Interested patients who were deemed safe to continue treatment had their dosing protocols adjusted according to the AAAAI practice parameter and CSACI Immunotherapy Manual recommendations [1, 2]. Nine patients (90%) chose to continue with SCIT, and one patient (10%) chose to discontinue therapy. The patient who chose to discontinue SCIT had been experiencing longstanding respiratory and cardiac symptoms not yet diagnosed, and shared decision-making was utilized to pause immunotherapy until his symptoms were further investigated. For the patients continuing SCIT, buildup was paused and SCIT was maintained at a lower dose until the relevant pollen season ended. As per CSACI guidelines, for patients who experienced severe anaphylactic reactions, the subsequent dose of allergen extract was reduced to 10% of the previous dose. For those who experienced mild systemic reactions, the subsequent dose was reduced to 50% [1]. All patients gradually returned to their previously prescribed maintenance dose without further adverse events during our analysis period.

## Discussion

Systemic reactions to SCIT occur in approximately 0.1% of injection visits, based on surveillance studies conducted between 2008 and 2016 in North America [4]. In a recent paper by Robertson et al. the rate of systemic reactions was 0.095% per injection in a referral-based private practice in Kitchener, Ontario [8]. Whereas the rate of systemic reactions to SCIT at this Canadian Allergy & Immunology clinic was 0.24% per injection. This rate is higher than the average rate reported, and almost half of the reactions that occurred were possibly avoidable. Thus, this QI initiative highlights the need for improved education on risk factors (Table 3) and improved screening for adherence.

**Table 3:** Summary of Identified Risk Factors for Patients with Systemic Reactions to SCIT

Risk factor	Number of patients
1. First injection from a new serum vial	4
2. Seasonal exacerbation of allergic disease	4
3. History of previous systemic reactions to SCIT	3
4. Sub-optimally controlled asthma	2
5. Omission of pre-medication	1
6. Exercise 1 h prior to injection	1
7. ACE-Inhibitor and NSAID usage coinciding with injection	1
8. Intramuscular administration	1

*ACE* Angiotensin converting enzyme, *NSAID* Nonsteroidal anti-inflammatory drug, *SCIT* Subcutaneous immunotherapy

The 2011 AAAAI practice parameters and 2016 CSACI Immunotherapy Manual provide recommendations for controlling risk factors in SCIT [1, 2]. Many of the recommendations, such as adherence to asthma maintenance therapy and reporting disease exacerbation, are the responsibility of the patient. Our clinic requires patients to sign a contractual agreement that includes these instructions and precautions prior to commencing immunotherapy. The cases highlighted in our QI initiative demonstrate the need for additional checkpoints; for example, the physician or nurse administering SCIT should screen patients for adequate asthma control, adherence to safety instructions, appropriate pre-medication, and any recent changes to medications or lifestyle before each injection. Using a standardized pre-injection screening questionnaire can ensure consistent screening for all patients. A reminder at each visit may help to stress the importance of adherence and atopic disease optimization for reducing the risk of systemic reactions.

We identified one possible case of error in administration, involving intramuscular rather than subcutaneous injection of extracts, as determined by landmarking on physical exam by the supervising physician. Although this is an uncommon event, certain patients may be at an increased risk for such an error, including those with limited subcutaneous tissue. This case emphasizes the importance of maintaining technical skills through continuing medical education. To further reduce the risk of intramuscular injection, ultrasound guidance, as proposed by Kim et al. could be used where resources permit to accurately determine subcutaneous tissue depth prior to injection [9]. More broadly, errors in administration, such as dosing errors and incorrect injection site, have been shown to contribute to almost one-third of reactions in a survey conducted by Amin et al. [10] In particular, dosing errors were reported by physicians to be the second most important contributor to reactions to SCIT [10]. Similarly, Aaronson and Gandhi surveyed allergists’ experiences with incorrect allergy injections, finding that 58% recalled a patient receiving an injection meant for another patient, and 74% recalled a patient receiving the incorrect dose at least once over a 5 year period [11]. These findings in part reflect human error, but also the environment in which they occur. Prevention strategies should evaluate systemic errors, with solutions targeting workplace guidelines and practices informed by patient safety literature. For example, it is important for the patient and clinic to have a log of the patient’s dosing protocol, and for nurses and physicians to verify doses before administration. Clinic staff should verify patient identity with at least two identifiers prior to any medical procedure, including at the time of immunotherapy administration [11].

All reactions occurred with the administration of the highest extract concentration (1:1 vol/vol), denoted in the Canadian community as vial 4. 40% of the reactions occurred after the first injection of a new serum vial, including one patient in the maintenance phase, and three patients in the build-up phase. Previous studies have shown that receiving an injection from a new vial is a risk factor for a systemic reaction [2]. It is expected that the quicker the increase in dose and the higher the allergen concentration, the greater the risk of a reaction. This finding emphasizes the importance of ensuring appropriate dose reduction with a new vial of SCIT.

We found that 50% of the reactions occurred during the build-up phase, with the highest extract concentration (vial 4). In a retrospective case control study, Mustafa et al. determined that 77.8% of systemic reactions occurred at a dose of 0.1 mL or higher of vial 4 [12]. A suggested dosing protocol from the 2016 CSACI Immunotherapy Manual recommends beginning vial 4 with a lower initial dose and a slower build-up phase compared to prior vials [1]. These guidelines leave room for doses and intervals between doses to be adjusted based on each patient’s tolerance and risk factors. Given the frequent implication of vial 4 in systemic reactions, it may be beneficial to consider adjusting the dosing protocol to begin with an even lower dose followed by smaller incremental increases for patients who are otherwise at an increased risk of systemic reactions to SCIT. Further research is needed to determine an optimal dosing protocol.

All ten reactions involved patients receiving SCIT for aeroallergens; no patients undergoing venom immunotherapy experienced a systemic reaction during the study period. This finding is noteworthy as our clinic uses a unique and customized venom immunotherapy protocol (Table 1) that includes an accelerated build-up phase. Rush protocols for venom immunotherapy that aim to achieve a maintenance dose in days to weeks have been demonstrated to be equally effective and safe when compared to conventional, slower protocols [13–15]. Unlike immunotherapy for aeroallergens [16], rush protocols for venom immunotherapy do not pose an increased risk for systemic reactions, and this is corroborated by our findings [13, 17, 18]. Note however that ultra-rush protocols for venom immunotherapy (achieving maintenance dosing in 4 h) have been associated with an increased risk for systemic reactions [19].

The majority of reactions (80%) involved a mixture of standardized and non-standardized extracts, and a minority (20%) involved standardized extracts only. Non-standardized extracts present the potential for an increased risk of systemic reactions due to variation in extract concentration and composition between batch preparations [2]. This risk may be mitigated by using standardized extracts whenever possible, with products currently available for five allergens (Hymenoptera venom, dust mite, cat, short ragweed pollen, and grass pollens) [1]. Additionally, we found that all reactions occurred with extracts that contained multiple allergens (lowest number of allergens per vial: two, highest: seven). Multiallergen SCIT is the most common practice among US allergists, as such the safety and efficacy of this approach is demonstrated in the AAAAI/ACAAI safety data [2, 4]. The use of multiallergen extracts in polysensitized patients was therefore felt to have limited contribution to patient risk profiles.

In analyzing individual extracts in patients with systemic reactions, we found that eight patients received cat dander extract, two received dog dander extract, and nine received grass pollen extract. These three aeroallergen extracts have been associated with an increased risk of systemic reactions to SCIT [12]. Notably, two patients did not have any obvious risk factors for systemic reaction; however, they had received injections containing grass at the time of their reaction. The significance of these allergens in patient risk stratification should be explored in future research.

Five patients had comorbid asthma, including two determined to have uncontrolled asthma at the time of their reaction. Recent analysis of the multi-year ACAAI/AAAAI National Surveillance Study identified uncontrolled asthma to be a leading risk factor for systemic reactions to SCIT, concluding that risk management strategies should target patients with asthma [20]. These findings emphasize the importance of routine screening for asthma symptoms and lung function, with a recommendation to withhold injections in patients with treatment refractory asthma, those exhibiting symptoms at the time of assessment, and/or evidence of recent decline on pulmonary function tests [20].

Four patients exhibited seasonal allergic rhinitis exacerbation that was not detected prior to administration. These reactions may have been prevented through a focused screening procedure, or through measured dose reduction during the peak pollen season [1]. SCIT administration during peak pollen season was the most important factor contributing to systemic reactions identified by Amin et al. reported in 46% of cases [10].

We identified three potential risk factors that have mixed or limited evidence within the literature. These include the omission of pre-medication, strenuous exercise prior to immunotherapy, and the concurrent use of an ACE inhibitor. The use of antihistamine premedication may reduce the frequency of systemic reaction in conventional and rush dosing protocols, with greater evidence to support its use with accelerated schedules [2]. The risk conferred by the omission of premedication is unknown; however, it poses a challenge in the interpretation of reactions and individual patient sensitivities. Strenuous exercise prior to receiving immunotherapy carries a theoretical risk of worsening asthma symptoms in susceptible patients. Suboptimal lung function around the time of injections may be misinterpreted as (or potentially promote) a systemic reaction is this population. The possibility of uncharacterized asthma should be considered prior to the initiation of SCIT, and the avoidance of heavy exercise around the time of injection should be discussed. The concurrent use of ACE inhibitors is a recognized risk factor for systemic reactions in venom immunotherapy, although there is some evidence to the contrary [2, 21]. It has not been characterized as a risk factor in patients receiving SCIT for aeroallergens; however, it carries a theoretical risk of promoting anaphylaxis through the impaired breakdown of vasoactive kinins [22].

Reactions to SCIT that occur more than 30 min post-injection are considered delayed reactions. In our QI initiative, most reactions happened within 30 min of injection. One occurred 40 min post-injection and another 1 h post-injection, making up 20% of the reactions. Existing literature varies in reports of delayed systemic reactions. In a large prospective study, 0.16% of injections were associated with delayed reactions [23]. Moreno et al. found that all reactions occurred within 30 min [24], while Rank et al. found that 48% of systemic reactions occurred after 30 min [25]. Therefore, it is important to have patients remain in the clinic for at least 30 min, and for even longer if the individual has a history of delayed reactions or is at increased risk of systemic reactions. Patients should be reminded to report delayed reactions to physicians, and physicians should ensure that events are clearly documented and appropriate changes to dosing protocols are made. Patients may also benefit from having a detailed plan created with healthcare professionals for managing systemic reactions that occur after leaving the clinic, which may include the availability of an epinephrine autoinjector at the discretion of the treating physician [2, 25].

The findings in this QI initiative reflect the real-world experience of allergists in a large academic institution over a one year period. Many of the risk factors identified are well described in the literature and may have been potentially avoided. Our work highlights a need for diligence in assessing individual risk factors on an ongoing basis, with attention to modifiable risks such as inadequate asthma control or varying sensitivities through relevant pollen seasons. Moreover, this work reflects a need for effective communication with patients, both to identify changes in patients’ health status and to inform patients on how to maintain best safety practices between injections.

**Fig. 1:**
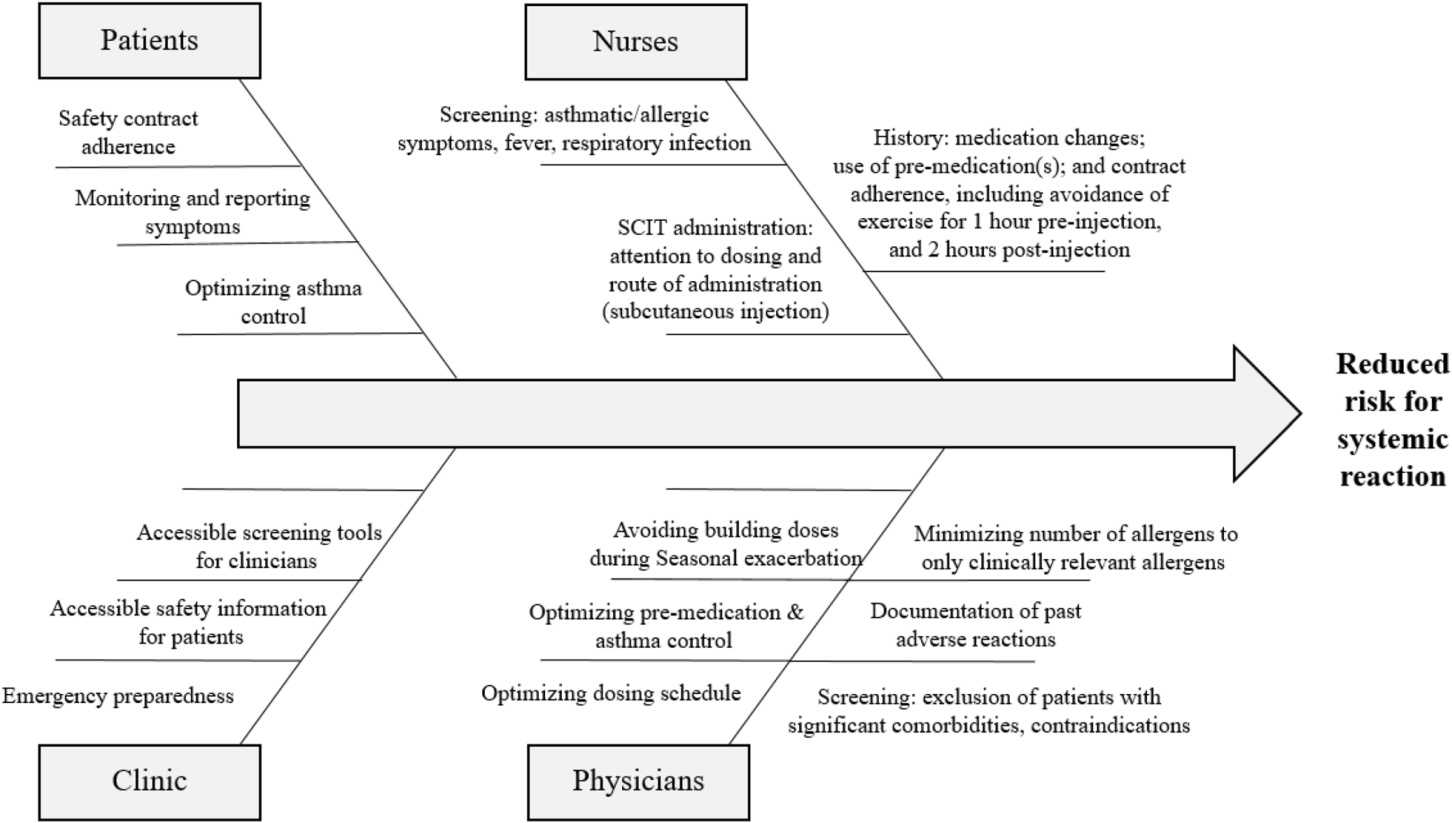
Systemic Reaction Quality Improvement Diagram. Analysis of factors which may be targeted to reduce the risk of systemic reactions. These identified factors can be divided into four categories: patient, nurse, clinic, and physician

A summary of our recommendations for quality improvement is provided in a diagram (Fig. 1). Our recommendations are presented under four headings (Patients, Nurses, Clinic, and Physicians) that serve as examples of individuals who may take responsibility for these actions. Patients take the responsibility of adhering to their safety contract, avoiding outlined contraindications, and reporting changes in their health status. Nurses serve an integral role in screening for symptomatic patients prior to injections, educating patients on the best safety practices, and ensuring safety in SCIT administration. Administrative staff in the clinic may support patients by providing screening tools for use prior to injection, take-home safety information for reference, and optimizing clinic flow to ensure adequate staffing and supplies for emergency situations. Physicians may use discretion in optimizing patients’ dosing schedules with consideration of their known risk factors, with particular attention to modifiable factors such as patients with symptomatic asthma and patients with seasonal exacerbation of their allergic disease.

Our QI initiative is limited by the sample size. Low rates of systemic reactions reflect the safety of SCIT for aeroallergens but provide limited data for analysis. The higher percentage of females over males among those with systemic reactions to SCIT (80% females vs 20% males) may be a product of the small sample size, and may reflect the female predominance (59%) in the overall cohort of patients undergoing SCIT during the analysis period. However, four retrospective studies have made similar observations [12, 26–28]. This QI initiative provides a snapshot of the rate of systemic reactions to SCIT at a large Canadian Allergy clinic when limited Canadian data is available. From these cases, we have identified modifiable risk factors for systemic reactions and provided suggestions for quality improvement in future practices.

## Conclusion

Our QI initiative of SCIT in a Canadian academic centre showed an incidence rate of systemic reactions requiring epinephrine administration of 0.24% of SCIT injections. Several risk factors were identified, including potentially avoidable errors that led to an increased risk for adverse reactions. For each risk factor identified, we proposed recommendations to improve patient safety using a QI model. We suggest that clinicians administering SCIT screen patients for treatment plan adherence prior to each injection. Extra precaution should be used with high risk patients, including highly sensitive patients and those with multiple risk factors for adverse reactions. When possible, these patients should have injection visits completed in hospital. Finally, we noted that all cases involved an adverse reaction to vial 4, containing the highest concentration of allergen extract. Further research into the dosing protocol and safety of high concentration preparations is needed. Lower doses and more gradual dose increase for the initial injections of vial 4 may be considered in high risk patients.

## Data Availability

All data generated and analyzed during this quality improvement project are included in this published article.

## Abbreviations

SCIT: Subcutaneous immunotherapy
ACE: Angiotensin converting enzyme
NSAID: Non-steroidal anti-inflammatory drug
CSACI: Canadian Society of Allergy and Clinical Immunology
AAAAI: American Academy of Allergy, Asthma, and Immunology
ACAAI: American College of Allergy, Asthma, and Immunology
EAACI: European Academy of Allergy and Clinical Immunology
WAO: World allergy organization
IM: Intramuscular
PO: Per os
SC: Subcutaneous
